# The genome sequence of the spider, *Parasteatoda lunata *(Clerck, 1757)

**DOI:** 10.12688/wellcomeopenres.19585.1

**Published:** 2023-06-22

**Authors:** Geoff Oxford, Natural History Museum Genome Acquisition Lab, Darwin Tree of Life Barcoding collective, Wellcome Sanger Institute Tree of Life programme, Wellcome Sanger Institute Scientific Operations: DNA Pipelines collective, Tree of Life Core Informatics collective, Darwin Tree of Life Consortium

**Affiliations:** 1 Department of Biology, University of York, York, England, YO10 5DD, UK

**Keywords:** Parasteatoda lunata, spider, genome sequence, chromosomal, Araneae

## Abstract

We present a genome assembly from an individual female *Parasteatoda lunata *(spider; Arthropoda; Arachnida; Araneae; Theridiidae). The genome sequence is 1,411.4 megabases in span. Most of the assembly is scaffolded into 12 chromosomal, including the X _1_ and X _2_ sex chromosomes. The mitochondrial genome has also been assembled and is 14.29 kilobases in length.

## Species taxonomy

Eukaryota; Metazoa; Eumetazoa; Bilateria; Protostomia; Ecdysozoa; Panarthropoda; Arthropoda; Chelicerata; Arachnida; Araneae; Araneomorphae; Entelegynae; Orbiculariae; Araneoidea; Theridiidae; Parasteatoda (Clerck, 1757) (NCBI:txid1871986).

## Background

The theridiid *Parasteatoda lunata* (formerly *Achaearanea lunata*) holds a special place in arachnological history in that it was one of the first spiders to be recorded at a specified locality anywhere in the world. The recorder was Martin Lister (1678) and the locality, Askham Wood, near York ( Parker & Harley, 1992:106–107).

*Parasteatoda lunata* is one of three *Parasteatoda* species found in the British Isles; the others are *P. simulans* and *P. tepidariorum*. The last species is now widely used as a model organism with which to study arachnid and, more generally, chelicerate development (for example, Hilbrant *et al.*, 2012; Oda & Akiyama-Oda, 2020). These, and three *Cryptachaea* species, comprise a group of British theridiids in which the abdomen is teardrop-shaped and distinctly upturned so that the anterior section appears much higher relative to the carapace, and the spinnerets point downwards ( Bee *et al.*, 2020).

*Parasteatoda lunata* builds a tangled framework of silken threads with sticky globules at the anchor points where the web meets substrate. These globules detain prey until the spider can strike. Typically, the webs are built in shaded, sheltered locations, often in deep crevices in tree trunks or in the cavities of I-shaped metal fence uprights where they are overshadowed by trees. The female always incorporates a leaf or other debris in the web to act as a shelter for her and her multiple grey/brown egg sacs. The species is common in the Midlands and south-eastern England but absent from Wales and Scotland. It seems to be extending its range northwards with the recent (re)colonisation of central Yorkshire – apparently the first records here since Lister’s time.

The genome of *Parasteatoda lunata* was sequenced as part of the Darwin Tree of Life Project, a collaborative effort to sequence all named eukaryotic species in the Atlantic Archipelago of Britain and Ireland. Here we present a chromosomally complete genome sequence for *Parasteatoda lunata* based on one female specimen from New Earswick, York. The *P. lunata* genome will provide an extremely useful comparison to that of the closely related *P. tepidariorum* ( Schwager *et al.*, 2017), as well as the genomes of more distantly related spiders in terms of the evolution of genome content and structure.

## Genome sequence report

**Figure 1.**
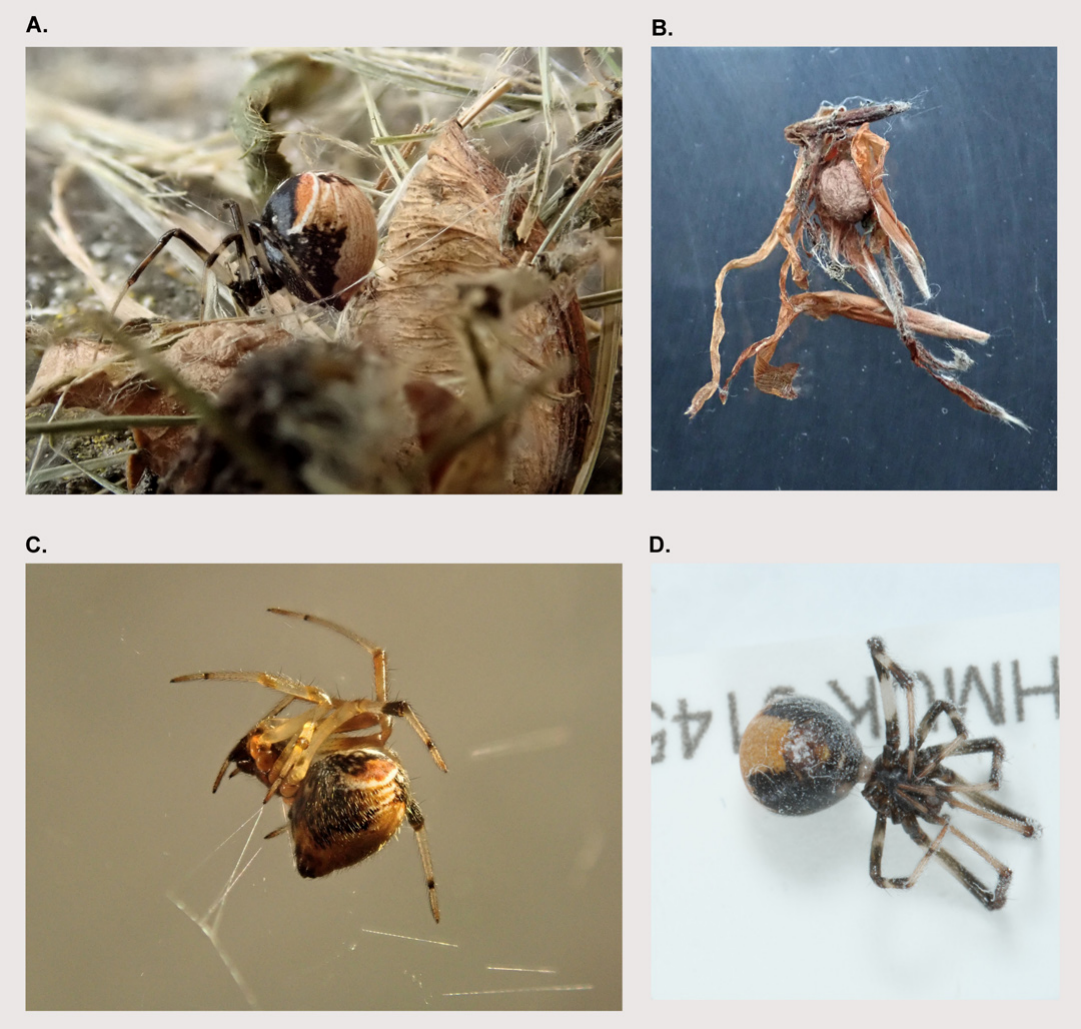
Photographs of *Parasteatoda lunata* individuals from the New Earswick population **A**. Female spider in retreat, **B**. Retreat and egg sac, **C**. Female spider in web, **D**. *P. lunata* (qqParLuna2) specimen during preservation and processing. (Photographs **A**– **C** by Geoff Oxford.)

The genome was sequenced from one female *Parasteatoda lunata* ( Figure 1) collected from Joseph Rowntree School, New Earswick, UK (53.99, –1.07). A total of 31-fold coverage in Pacific Biosciences single-molecule HiFi long reads was generated. Primary assembly contigs were scaffolded with chromosome conformation Hi-C data. Manual assembly curation corrected 64 missing joins or mis-joins and removed 4 haplotypic duplications, reducing the scaffold number 8.38%, and increasing the scaffold N50 by 8.99%.

**Figure 2.**
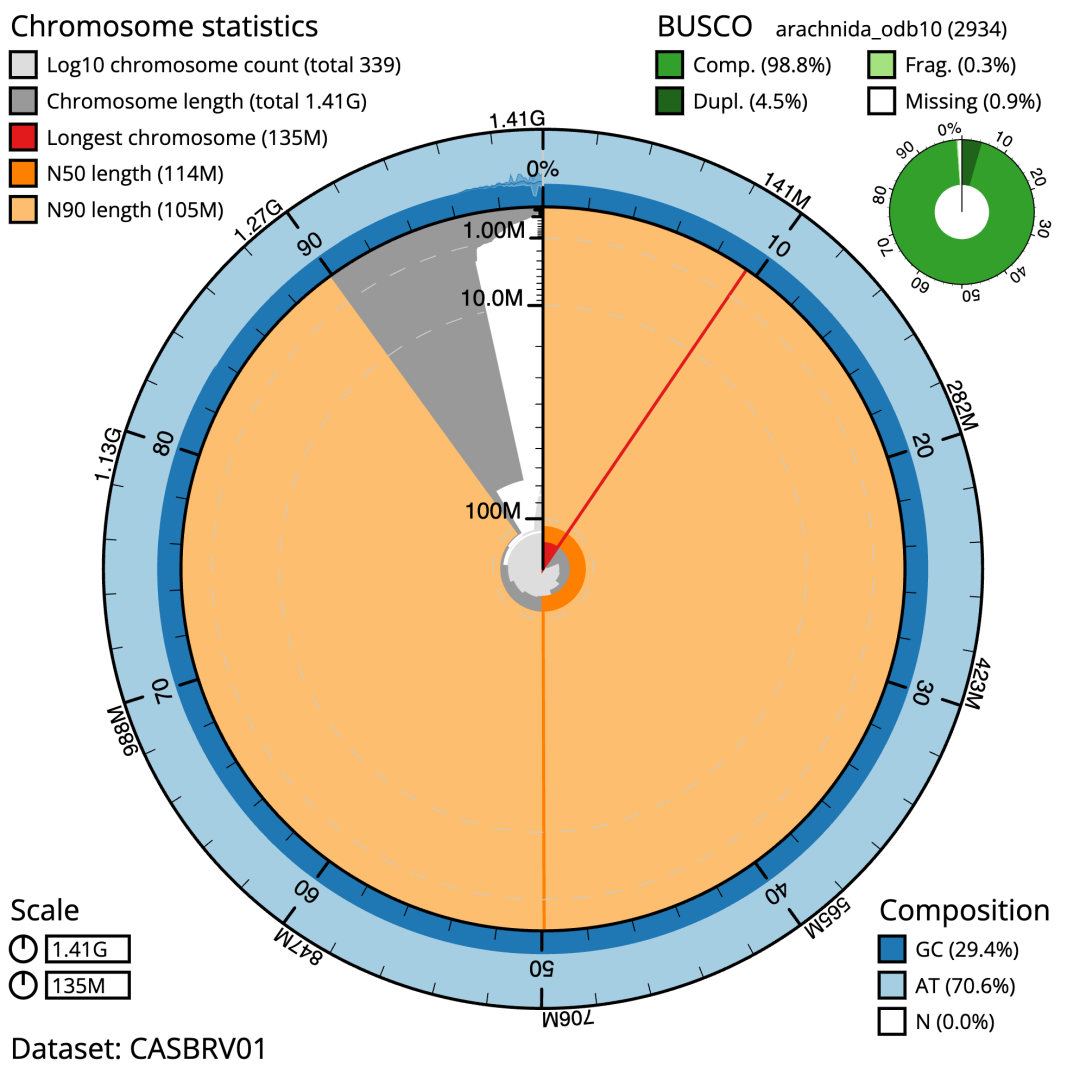
Genome assembly of *Parasteatoda lunata*, qqParLuna2.1: metrics. The BlobToolKit Snailplot shows N50 metrics and BUSCO gene completeness. The main plot is divided into 1,000 size-ordered bins around the circumference with each bin representing 0.1% of the 1,411,378,570 bp assembly. The distribution of scaffold lengths is shown in dark grey with the plot radius scaled to the longest scaffold present in the assembly (134,647,540 bp, shown in red). Orange and pale-orange arcs show the N50 and N90 scaffold lengths (114,314,067 and 104,696,160 bp), respectively. The pale grey spiral shows the cumulative scaffold count on a log scale with white scale lines showing successive orders of magnitude. The blue and pale-blue area around the outside of the plot shows the distribution of GC, AT and N percentages in the same bins as the inner plot. A summary of complete, fragmented, duplicated and missing BUSCO genes in the arachnida_odb10 set is shown in the top right. An interactive version of this figure is available at https://blobtoolkit.genomehubs.org/view/qqParLuna2.1/dataset/CASBRV01/snail.

The final assembly has a total length of 1,411.4 Mb in 338 sequence scaffolds with a scaffold N50 of 114.3 Mb ( Table 1). Most (96.61%) of the assembly sequence was assigned to 12 chromosomal-level scaffolds, representing 10 autosomes and the X _1_ and X _2_ sex chromosomes. Chromosome-scale scaffolds confirmed by the Hi-C data are named in order of size ( Figure 2– Figure 5; Table 2). The X _1_ and X _2_ chromosomes were identified based on synteny with *Metellina segmentata* (GCA_947359465.1) ( Henriques & Sivell, 2023). While not fully phased, the assembly deposited is of one haplotype. Contigs corresponding to the second haplotype have also been deposited. The mitochondrial genome was also assembled and can be found as a contig within the multifasta file of the genome submission.

**Table 1.** Genome data for *Parasteatoda lunata*, qqParLuna2.1.

Project accession data		
Assembly identifier	qqParLuna2.1	
Species	*Parasteatoda lunata*	
Specimen	qqParLuna2	
NCBI taxonomy ID	1871986	
BioProject	PRJEB57892	
BioSample ID	SAMEA110034684	
Isolate information	qqParLuna2, female: whole organism (DNA sequencing) qqParLuna1, female: whole organism (Hi-C scaffolding)	
Assembly metrics *		*Benchmark*
Consensus quality (QV)	59.4	*≥ 50*
*k*-mer completeness	100%	*≥ 95%*
BUSCO **	C:98.8%[S:94.3%,D:4.5%], F:0.3%,M:0.9%,n:2,934	*C ≥ 95%*
Percentage of assembly mapped to chromosomes	96.61%	*≥ 95%*
Sex chromosomes	X _1_ and X _2_	*localised homologous pairs*
Organelles	Mitochondrial genome assembled	*complete single alleles*
Raw data accessions		
PacificBiosciences SEQUEL II	ERR10662017, ERR10662018	
Hi-C Illumina	ERR10614872	
Genome assembly		
Assembly accession	GCA_949128135.1	
*Accession of alternate haplotype*	GCA_949128125.1	
Span (Mb)	1,411.4	
Number of contigs	934	
Contig N50 length (Mb)	3.6	
Number of scaffolds	338	
Scaffold N50 length (Mb)	114.3	
Longest scaffold (Mb)	134.7	

* Assembly metric benchmarks are adapted from column VGP-2020 of “Table 1: Proposed standards and metrics for defining genome assembly quality” from ( Rhie *et al.*, 2021). ** BUSCO scores based on the arachnida_odb10 BUSCO set using v5.3.2. C = complete [S = single copy, D = duplicated], F = fragmented, M = missing, n = number of orthologues in comparison. A full set of BUSCO scores is available at https://blobtoolkit.genomehubs.org/view/qqParLuna2.1/dataset/CASBRV01/busco.

**Figure 3.**
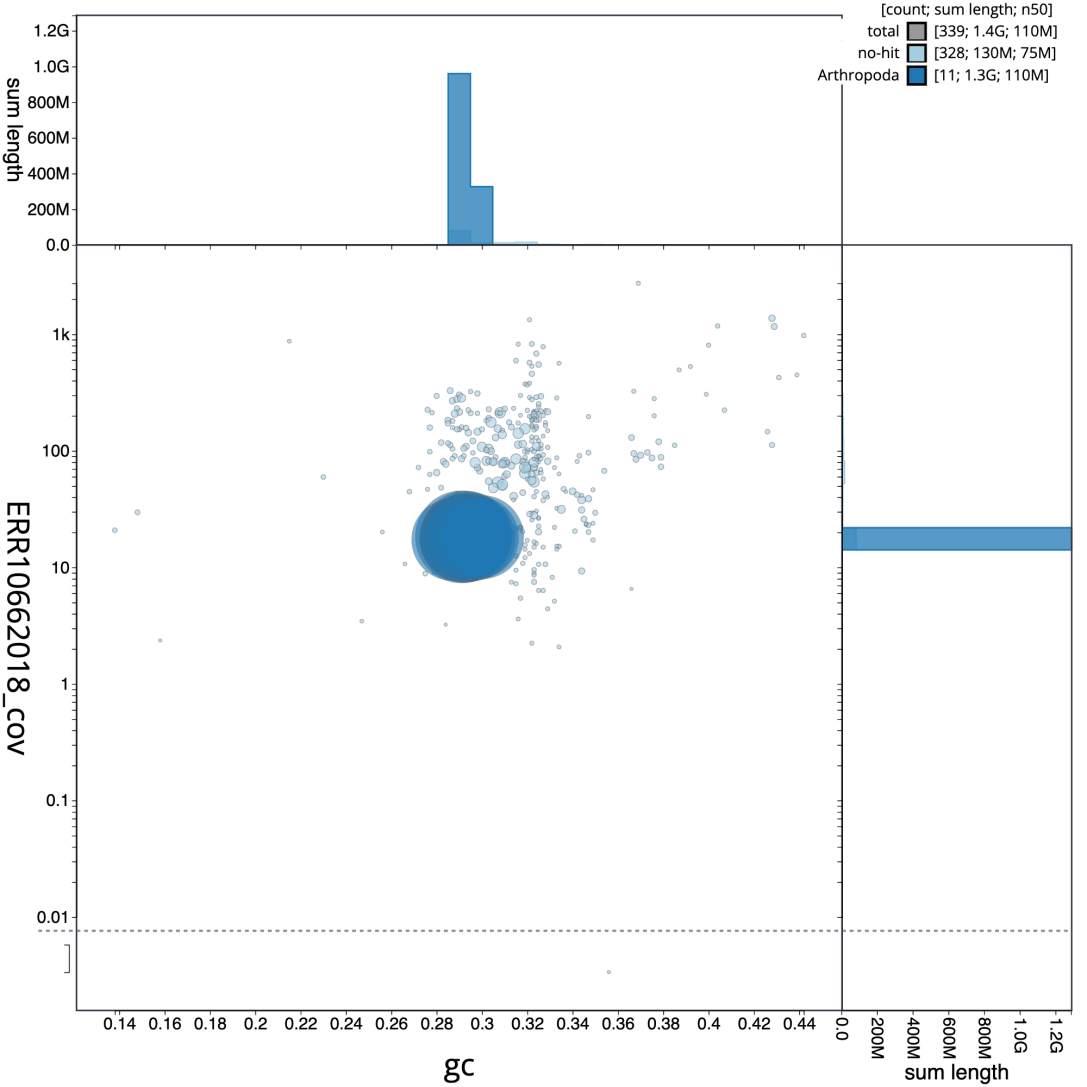
Genome assembly of *Parasteatoda lunata*, qqParLuna2.1: BlobToolKit GC-coverage plot. Scaffolds are coloured by phylum. Circles are sized in proportion to scaffold length. Histograms show the distribution of scaffold length sum along each axis. An interactive version of this figure is available at https://blobtoolkit.genomehubs.org/view/qqParLuna2.1/dataset/CASBRV01/blob.

**Figure 4.**
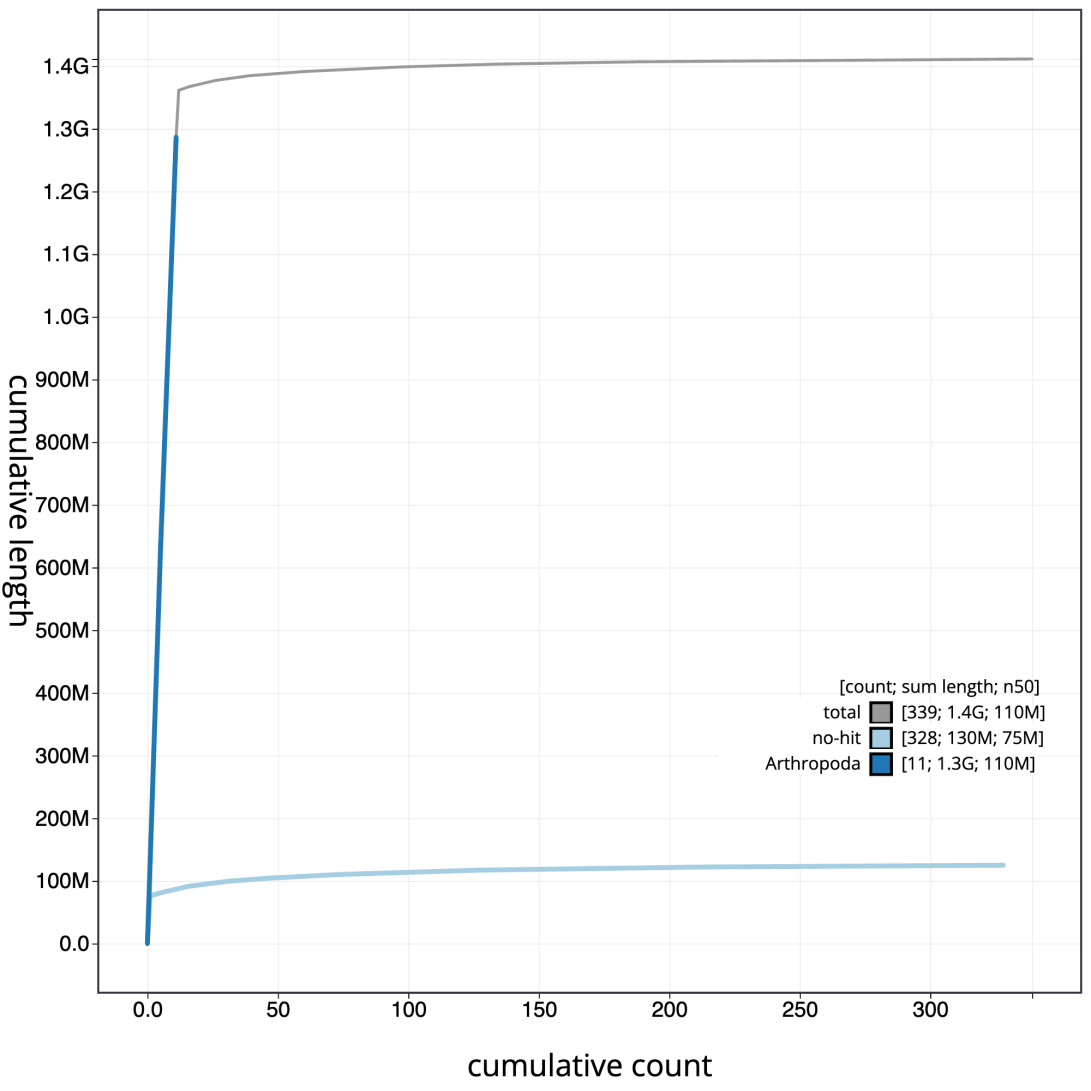
Genome assembly of *Parasteatoda lunata*, qqParLuna2.1: BlobToolKit cumulative sequence plot. The grey line shows cumulative length for all scaffolds. Coloured lines show cumulative lengths of scaffolds assigned to each phylum using the buscogenes taxrule. An interactive version of this figure is available at https://blobtoolkit.genomehubs.org/view/qqParLuna2.1/dataset/CASBRV01/cumulative.

**Figure 5.**
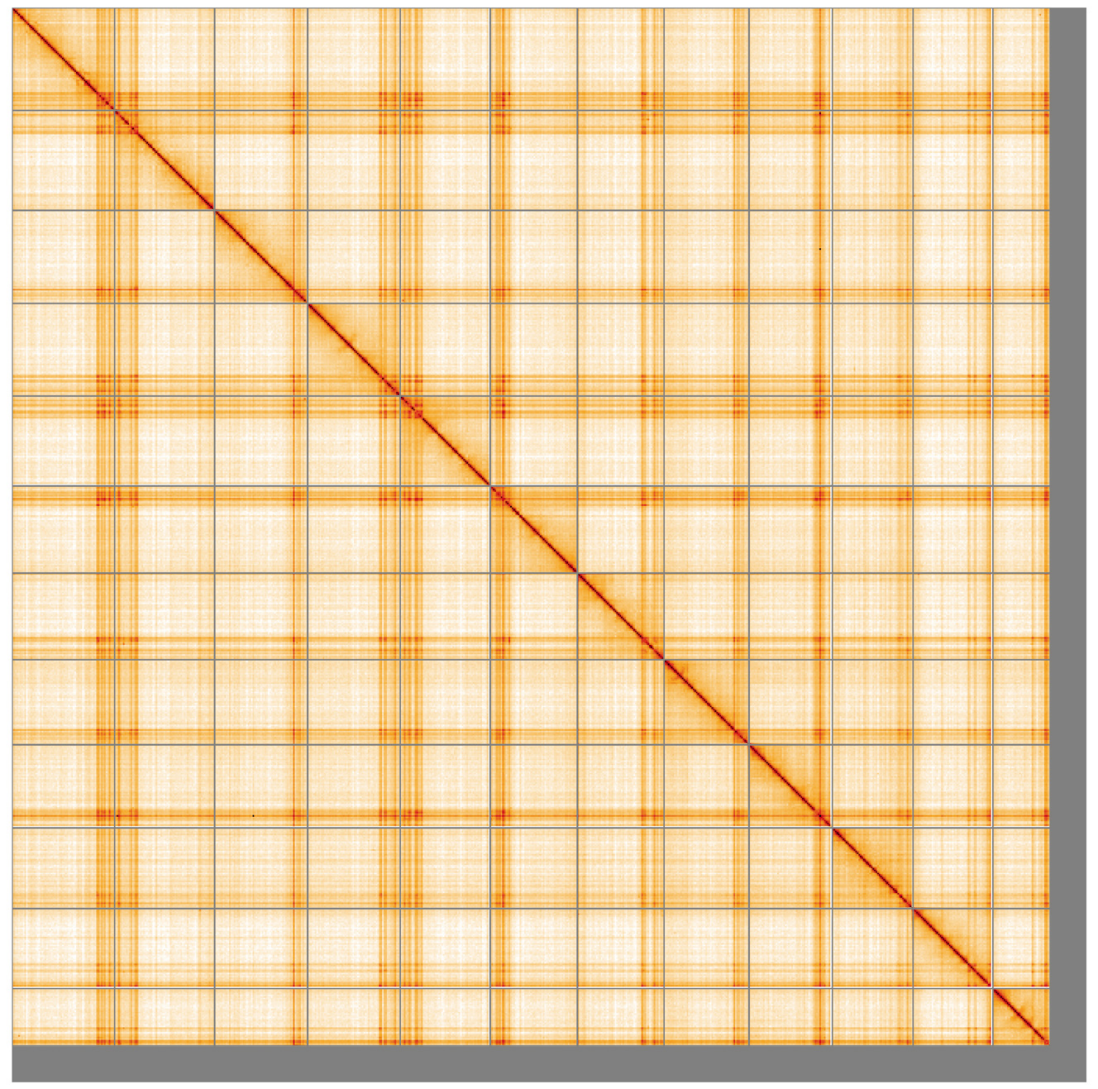
Genome assembly of *Parasteatoda lunata*, qqParLuna2.1: Hi-C contact map of the qqParLuna2.1 assembly, visualised using HiGlass. Chromosomes are shown in order of size from left to right and top to bottom. An interactive version of this figure may be viewed at https://genome-note-higlass.tol.sanger.ac.uk/l/?d=UJaCEon1R-OQIOKvaJNE0g.

**Table 2.** Chromosomal pseudomolecules in the genome assembly of *Parasteatoda lunata*, qqParLuna2.

INSDC accession	Chromosome	Length (Mb)	GC%
OX421970.1	1	134.65	29.0
OX421971.1	2	131.09	29.5
OX421972.1	3	122.06	29.0
OX421973.1	4	121.47	29.0
OX421974.1	5	117.94	29.5
OX421975.1	6	114.31	29.5
OX421976.1	7	113.5	29.5
OX421977.1	8	111.55	29.5
OX421978.1	9	109.19	30.0
OX421979.1	10	105.89	30.0
OX421980.1	X _1_	104.7	28.5
OX421981.1	X _2_	75.08	29.0
OX421982.1	MT	0.01	21.5

The estimated Quality Value (QV) of the final assembly is 59.4 with *k*-mer completeness of 100%, and the assembly has a BUSCO v5.3.2 completeness of 98.8% (single = 94.3%, duplicated = 4.5%), using the arachnida_odb10 reference set ( *n* = 2,934).

Metadata for specimens, spectral estimates, sequencing runs, contaminants and pre-curation assembly statistics can be found at https://links.tol.sanger.ac.uk/species/1871986.

## Methods

### Sample acquisition and nucleic acid extraction

Two female *Parasteatoda lunata* specimens used for genome sequencing (specimen ID NHMUK014537466, individual qqParLuna2) and Hi-C scaffolding (specimen ID NHMUK014537472, individual qqParLuna1) were collected from Joseph Rowntree School, New Earswick, England (latitude 53.99, longitude –1.07) on 2021-06-03. The specimens were collected and identified by Geoff Oxford (University of York) and dry frozen (–80°C).

DNA was extracted at the Tree of Life laboratory, Wellcome Sanger Institute (WSI). The qqParLuna2 sample was weighed and dissected on dry ice. Whole organism tissue was disrupted using a Nippi Powermasher fitted with a BioMasher pestle. High molecular weight (HMW) DNA was extracted using the Qiagen MagAttract HMW DNA extraction kit. HMW DNA was sheared into an average fragment size of 12–20 kb in a Megaruptor 3 system with speed setting 30. Sheared DNA was purified by solid-phase reversible immobilisation using AMPure PB beads with a 1.8X ratio of beads to sample to remove the shorter fragments and concentrate the DNA sample. The concentration of the sheared and purified DNA was assessed using a Nanodrop spectrophotometer and Qubit Fluorometer and Qubit dsDNA High Sensitivity Assay kit. Fragment size distribution was evaluated by running the sample on the FemtoPulse system.

### Sequencing

Pacific Biosciences HiFi circular consensus DNA sequencing libraries were constructed according to the manufacturers’ instructions. DNA sequencing was performed by the Scientific Operations core at the WSI on a Pacific Biosciences SEQUEL II (HiFi) instrument. Hi-C data were also generated from whole organism tissue of qqParLuna1 using the Arima2 kit and sequenced on the Illumina NovaSeq 6000 instrument.

### Genome assembly, curation and evaluation

Assembly was carried out with Hifiasm ( Cheng *et al.*, 2021) and haplotypic duplication was identified and removed with purge_dups ( Guan *et al.*, 2020). The assembly was then scaffolded with Hi-C data ( Rao *et al.*, 2014) using YaHS ( Zhou *et al.*, 2023). The assembly was checked for contamination and corrected as described previously ( Howe *et al.*, 2021). Manual curation was performed using HiGlass ( Kerpedjiev *et al.*, 2018) and Pretext ( Harry, 2022). The mitochondrial genome was assembled using MitoHiFi ( Uliano-Silva *et al.*, 2022), which runs MitoFinder ( Allio *et al.*, 2020) or MITOS ( Bernt *et al.*, 2013) and uses these annotations to select the final mitochondrial contig and to ensure the general quality of the sequence.

A Hi-C map for the final assembly was produced using bwa-mem2 ( Vasimuddin *et al.*, 2019) in the Cooler file format ( Abdennur & Mirny, 2020). To assess the assembly metrics, the *k*-mer completeness and QV consensus quality values were calculated in Merqury ( Rhie *et al.*, 2020). This work was done using Nextflow ( Di Tommaso *et al.*, 2017) DSL2 pipelines “sanger-tol/readmapping” ( Surana *et al.*, 2023a) and “sanger-tol/genomenote” ( Surana *et al.*, 2023b). The genome was analysed within the BlobToolKit environment ( Challis *et al.*, 2020) and BUSCO scores ( Manni *et al.*, 2021; Simão *et al.*, 2015) were calculated.

Table 3 contains a list of relevant software tool versions and sources.

**Table 3.** Software tools: versions and sources.

Software tool	Version	Source
BlobToolKit	4.1.5	https://github.com/blobtoolkit/blobtoolkit
BUSCO	5.3.2	https://gitlab.com/ezlab/busco
Hifiasm	0.16.1-r375	https://github.com/chhylp123/hifiasm
HiGlass	1.11.6	https://github.com/higlass/higlass
Merqury	MerquryFK	https://github.com/thegenemyers/MERQURY.FK
MitoHiFi	2	https://github.com/marcelauliano/MitoHiFi
PretextView	0.2	https://github.com/wtsi-hpag/PretextView
purge_dups	1.2.3	https://github.com/dfguan/purge_dups
sanger-tol/genomenote	v1.0	https://github.com/sanger-tol/genomenote
sanger-tol/readmapping	1.1.0	https://github.com/sanger-tol/readmapping/tree/1.1.0
YaHS	1.2a	https://github.com/c-zhou/yahs

### Wellcome Sanger Institute – Legal and Governance

The materials that have contributed to this genome note have been supplied by a Darwin Tree of Life Partner. The submission of materials by a Darwin Tree of Life Partner is subject to the **‘Darwin Tree of Life Project Sampling Code of Practice’**, which can be found in full on the Darwin Tree of Life website here. By agreeing with and signing up to the Sampling Code of Practice, the Darwin Tree of Life Partner agrees they will meet the legal and ethical requirements and standards set out within this document in respect of all samples acquired for, and supplied to, the Darwin Tree of Life Project.

Further, the Wellcome Sanger Institute employs a process whereby due diligence is carried out proportionate to the nature of the materials themselves, and the circumstances under which they have been/are to be collected and provided for use. The purpose of this is to address and mitigate any potential legal and/or ethical implications of receipt and use of the materials as part of the research project, and to ensure that in doing so we align with best practice wherever possible. The overarching areas of consideration are and

- Ethical review of provenance and sourcing of the material
- Legality of collection, transfer and use (national and international)

Each transfer of samples is further undertaken according to a Research Collaboration Agreement or Material Transfer Agreement entered into by the Darwin Tree of Life Partner, Genome Research Limited (operating as the Wellcome Sanger Institute), and in some circumstances other Darwin Tree of Life collaborators.

## Data Availability

European Nucleotide Archive: *Parasteatoda lunata*. Accession number PRJEB57892; https://identifiers.org/ena.embl/PRJEB57892. ( Wellcome Sanger Institute, 2023) The genome sequence is released openly for reuse. The *Parasteatoda lunata* genome sequencing initiative is part of the Darwin Tree of Life (DToL) project. All raw sequence data and the assembly have been deposited in INSDC databases. The genome will be annotated using available RNA-Seq data and presented through the Ensembl pipeline at the European Bioinformatics Institute. Raw data and assembly accession identifiers are reported in Table 1.

## Author information

Members of the Natural History Museum Genome Acquisition Lab are listed here: https://doi.org/10.5281/zenodo.4790042.

Members of the Darwin Tree of Life Barcoding collective are listed here: https://doi.org/10.5281/zenodo.4893703.

Members of the Wellcome Sanger Institute Tree of Life programme are listed here: https://doi.org/10.5281/zenodo.4783585.

Members of Wellcome Sanger Institute Scientific Operations: DNA Pipelines collective are listed here: https://doi.org/10.5281/zenodo.4790455.

Members of the Tree of Life Core Informatics collective are listed here: https://doi.org/10.5281/zenodo.5013541.

Members of the Darwin Tree of Life Consortium are listed here: https://doi.org/10.5281/zenodo.4783558.
