# Supplementary material for: The genome sequence of the spider, Parasteatoda lunata (Clerck, 1757)
Source: Wellcome Open Res. 2023 Jun 22;8:271. [Version 1] doi: 10.12688/wellcomeopenres.19585.1 (PMC10521088; doi:10.12688/wellcomeopenres.19585.1)
Supplement: Peer review history

# Reviewer report: Reviewer response for version 1

**DOI:** 10.21956/wellcomeopenres.21695.r65974

**Published:** 2023-09-27

**Authors:** Stephane Rombauts

**Affiliations:** 1 Department of Plant Biotechnology and Bioinformatics, Ghent University, Ghent, Belgium

The genome reported here on the spider Parasteatoda lunata, not only contributes to the Tree of Life project, from the British Isles, but worldwide, for a better understanding of chelicerates and their ability to produce silk, a research topic in material sciences. Therefore, this genome will contribute well beyond the genome only.

Are sufficient details of methods and materials provided to allow replication by others?

Yes

Is the rationale for creating the dataset(s) clearly described?

Yes

Are the datasets clearly presented in a useable and accessible format?

Yes

Are the protocols appropriate and is the work technically sound?

Yes

Reviewer Expertise:

Bioinformatics, focused on genomics, from HMW gDNA extraction to sequencing, assembly, and annotation. Mainly for plants, but also for arthropods.

I confirm that I have read this submission and believe that I have an appropriate level of expertise to confirm that it is of an acceptable scientific standard.

# Reviewer report: Reviewer response for version 1

**DOI:** 10.21956/wellcomeopenres.21695.r65982

**Published:** 2023-09-11

**Authors:** Jesper Bechsgaard

**Affiliations:** 1 Department of Biology, Aarhus University, Aarhus, Denmark

This data note described the sequencing of the reference genome of *Parasteatoda lunata*. I see a value of using this reference genome in evo-devo, but would appreciate if the authors could be more specific about how they see it can be used in comparison to the related species that already have reference genomes.

Are sufficient details of methods and materials provided to allow replication by others?

Yes

Is the rationale for creating the dataset(s) clearly described?

Partly

Are the datasets clearly presented in a useable and accessible format?

Yes

Are the protocols appropriate and is the work technically sound?

Yes

Reviewer Expertise:

Population genetics, molecular evolution

I confirm that I have read this submission and believe that I have an appropriate level of expertise to confirm that it is of an acceptable scientific standard.

# Reviewer report: Reviewer response for version 1

**DOI:** 10.21956/wellcomeopenres.21695.r61013

**Published:** 2023-07-04

**Authors:** Zhi-Sheng Zhang

**Affiliations:** 1 Key Laboratory of Eco-environments in Three Gorges Reservoir Region (Ministry of Education), School of Life Sciences, Southwest University, Chongqing, China

This article provide a high quality of the genome assembly of the spider, *Parasteatoda lunata. *The rationale for creating the dataset(s) is clearly described. The protocols is appropriate and the work technically is sound. The methods and materials is provided to allow replication by others. And the datasets is clearly presented in a useable and accessible format.

Are sufficient details of methods and materials provided to allow replication by others?

Yes

Is the rationale for creating the dataset(s) clearly described?

Yes

Are the datasets clearly presented in a useable and accessible format?

Yes

Are the protocols appropriate and is the work technically sound?

Yes

Reviewer Expertise:

Diversity and ecology of spiders from South China; Systematics, Phylogeny and biogeography of several spider groups, such as Lycosidae, Agelenidae, Dictynidae, Hahniidae, Amaurobiidae, Titanoecidae, etc.; Adapation of some spiders in different habitats; Spider silk.

I confirm that I have read this submission and believe that I have an appropriate level of expertise to confirm that it is of an acceptable scientific standard.
